# Fabrication and Thermoelectric Characterization of Transition Metal Silicide-Based Composite Thermocouples

**DOI:** 10.3390/s18113759

**Published:** 2018-11-03

**Authors:** Gunes A. Yakaboylu, Rajalekshmi C. Pillai, Katarzyna Sabolsky, Edward M. Sabolsky

**Affiliations:** Department of Mechanical and Aerospace Engineering, West Virginia University, Morgantown, WV 26506, USA; gayakaboylu@mix.wvu.edu (G.A.Y.); pillai.rajalekshmi200@gmail.com (R.C.P.); kathy.sabolsky@mail.wvu.edu (K.S.)

**Keywords:** metal silicide, alumina, ceramic composite, thermocouple, thermoelectric, high temperature, harsh environment, Seebeck coefficient

## Abstract

Metal silicide-based thermocouples were fabricated by screen printing thick films of the powder compositions onto alumina tapes followed by lamination and sintering processes. The legs of the embedded thermocouples were composed of composite compositions consisting of MoSi_2_, WSi_2_, ZrSi_2_, or TaSi_2_ with an additional 10 vol % Al_2_O_3_ to form a silicide–oxide composite. The structural and high-temperature thermoelectric properties of the composite thermocouples were examined using X-ray diffraction, scanning electron microscopy and a typical hot–cold junction measurement technique. MoSi_2_-Al_2_O_3_ and WSi_2_-Al_2_O_3_ composites exhibited higher intrinsic Seebeck coefficients (22.2–30.0 µV/K) at high-temperature gradients, which were calculated from the thermoelectric data of composite//Pt thermocouples. The composite thermocouples generated a thermoelectric voltage up to 16.0 mV at high-temperature gradients. The MoSi_2_-Al_2_O_3_//TaSi_2_-Al_2_O_3_ thermocouple displayed a better performance at high temperatures. The Seebeck coefficients of composite thermocouples were found to range between 20.9 and 73.0 µV/K at a temperature gradient of 1000 °C. There was a significant difference between the calculated and measured Seebeck coefficients of these thermocouples, which indicated the significant influence of secondary silicide phases (e.g., Mo_5_Si_3_, Ta_5_Si_3_) and possible local compositional changes on the overall thermoelectric response. The thermoelectric performance, high sensitivity, and cost efficiency of metal silicide–alumina ceramic composite thermocouples showed promise for high-temperature and harsh-environment sensing applications.

## 1. Introduction

Accurate, real-time, and reliable temperature measurements are crucial for safer and more efficient operation in many industrial applications, such as power generation, coal gasification, and metal and glass manufacturing [1,2]. Many of these systems operate at temperatures reaching 1500–1650 °C, with possible extreme physical conditions such as highly redox atmospheres, and corrosive liquids and gases, which present additional challenges [3,4]. Therefore, there is an increasing demand for advanced sensing materials that are capable of measuring temperatures under harsh environments.

Noble metal and metal alloy thermocouples (e.g., Pt-Rh//Pt, chromel//alumel) have been extensively used in high-temperature sensing over the years. However, these thermocouples are proven to be incapable of withstanding increasingly harsh conditions due to some limitations, such as selective oxidation in air or oxygen atmospheres (e.g., oxidation of Rh at ~600–800 °C), and detrimental microstructural and compositional changes due to chemical reactions with present liquid/gas species [3,5,6]. These issues result in degradation of such thermocouples and large drift in thermoelectric output over time. It is reported that the lifetime of thin-film thermocouples made of noble metals could be only less than a minute at 1500 °C in oxidizing conditions [7]. In addition, their relatively lower thermoelectric voltage and sensitivity (low Seebeck coefficients) also do not satisfy the requirements for high-temperature and harsh-environment applications [5,8].

Therefore, ceramic-based (or non-metal-based) thermocouples have recently attracted attention as alternative materials for high-temperature sensing due to their high melting temperatures, chemical and thermal stability at high temperatures, oxidation resistance, and high thermoelectric output [3,7,8]. Research studies have been mostly conducted on various ceramic materials, such as carbides (e.g., TaC, TiC), silicides (e.g., MoSi_2_, TaSi_2_), and conductive oxides (e.g., In_2_O_3_, In_2_O_3_:SnO_2_, ZnO), to achieve high-temperature thermocouples with enhanced sensitivity, oxidation resistance, and overall thermoelectric output [1,5,6,9]. It is reported that thin-film thermocouples made of conductive oxides could exhibit high thermoelectric output voltage, thermal stability, and oxidation resistance, but that their melting points are significantly lower with respect to that of most silicides and carbides [3,7,8,10]. Another drawback of the mostly studied In_2_O_3_:SnO_2_-(ITO) and indium oxide-based thermocouples is the known strong volatilization effects of these oxides at high temperatures [11]. This volatilization highly lowers their service life under harsh environments, and also limits their operation range to temperatures below ~1200 °C. Both silicides and carbides are known as promising high-temperature ceramic materials with metallic-type electrical properties for use as advanced sensing materials. Although carbides have substantially higher melting points than the silicides, their low oxidation temperatures (~800–1200 °C) are considered as a major problem [7,12]. Among all these high-temperature ceramics, silicides show more promising features for use in extreme high temperatures and harsh conditions due to the combination of their sufficient melting temperatures (~1500–2370 °C), higher oxidation temperatures (1200–1650 °C), hot corrosion resistance, chemical/thermal stability, wide range of thermoelectric power, and ability of forming a protective surface layer for further passivation [3,6,7,13]. In a study performed by Kreider [6], the thermoelectric performance and stability of TiSi_2_-, MoSi_2_-, WSi_2_-, and TaSi_2_-based thin-film thermocouples were examined at high temperatures. MoSi_2_ and TiSi_2_ thermocouples revealed higher thermoelectric performance with 19.4–68.6 µV/°C and 6.8–26.9 mV at 500 °C, and they revealed excellent thermoelectric and thermal stability up to 1200 °C in air atmosphere due to a protective silicon oxide layer formation. The high-temperature thermoelectric performance of CrSi_2_//Pt thin-film thermocouple was also examined in air, where it exhibited ~50.0 mV thermoelectric voltage at 500 °C [7]. However, the formation of a protective silica layer on the surface of silicide thin films (e.g., MoSi_2_, CrSi_2_) may be a limiting factor for their service life and long-term performance in high-temperature oxidizing conditions, since the consumption of silicon in the thin film may cause compositional changes and drift in the sensor output [3,12]. The fabrication and thermoelectric properties of embedded MoSi_2_- and WSi_2_-based ceramic composite thick-film thermocouples with the addition of Al_2_O_3_ particles were reported in the authors’ previous study [14]. Thick-film composite thermocouples showed highly stable thermoelectric performance with 14.1–17.5 mV and 10.3–31.3 µV/K at 1000 °C. Therefore, the fabrication of thick-film silicide thermocouples could be highly beneficial for enhanced thermoelectric performance and stability.

It is evident that metal silicides and their composite forms have great potential for accurate and real-time high-temperature measurements under oxidizing and extreme harsh environments. However, surprisingly, there are limited studies on the fabrication and thermoelectric characterization of both thin- and thick-film ceramic composite thermocouples made of various metal silicides. In this study, metal silicide (MoSi_2_, WSi_2_, ZrSi_2_, TaSi_2_)-based ceramic composite thermocouples were fabricated with the addition of alumina (Al_2_O_3_) particles, and these materials were embedded into alumina preforms based on the processing route developed in the authors’ previous study [14]. The purpose of using alumina particles, and the embedding of the thick-film legs of the thermocouples, was to lower the grain growth of the silicides and to improve oxidation resistance at high temperatures. Composite//platinum (Pt) thermocouples were additionally fabricated to better understand the thermoelectric properties of composites and composite//composite thermocouples. Their phase stability, microstructures, and thermoelectric properties at high temperatures were characterized and discussed.

## 2. Materials and Methods

### 2.1. Fabrication of Ceramic Composite Thermocouples

MoSi_2_ (99.5%), WSi_2_ (99.5%), TaSi_2_ (99.0%), and ZrSi_2_ (99.5%) commercial powders, all purchased from Alfa Aesar (Tewksbury, MA, USA), were used as starting metal silicides in this study for preparation of the ceramic composite mixtures and fabrication of the composite thermocouples. In addition, Al_2_O_3_ powders (99.8%, SSA: 8.6 m^2^/g) were purchased from Almatis (Leetsdale, PA, USA). Prior to the fabrication of the ceramic composite thick-film thermocouples, MoSi_2_-Al_2_O_3_, WSi_2_-Al_2_O_3_, TaSi_2_-Al_2_O_3_, and ZrSi_2_-Al_2_O_3_ composite powder mixtures, all having 90 vol % of the starting metal silicides and 10 vol % alumina, were prepared by ball-milling in ethanol for 24 h using yttria-stabilized zirconia milling media. For simplicity, the composition designation of [90–10] was used to identify the volume percentage of the composites (e.g., [90–10] TaSi_2_-Al_2_O_3_ indicates 90 vol % TaSi_2_ and 10 vol % Al_2_O_3_). After drying at 80 °C in a vacuum oven, they were screened using a sieve with 44 µm openings (325 mesh). Metal silicide–alumina composite powders were individually mixed with an organic vehicle (63-2, Johnson Matthey, Wayne, PA, USA) and ultrasonicated for preparing the required composite inks for the screen printing of ceramic composite thermocouples on alumina substrates. Platinum inks were also prepared using the same procedure for the fabrication of composite//Pt thermocouples, which were used to characterize the intrinsic thermoelectric performance of each composite.

**Figure 1:**
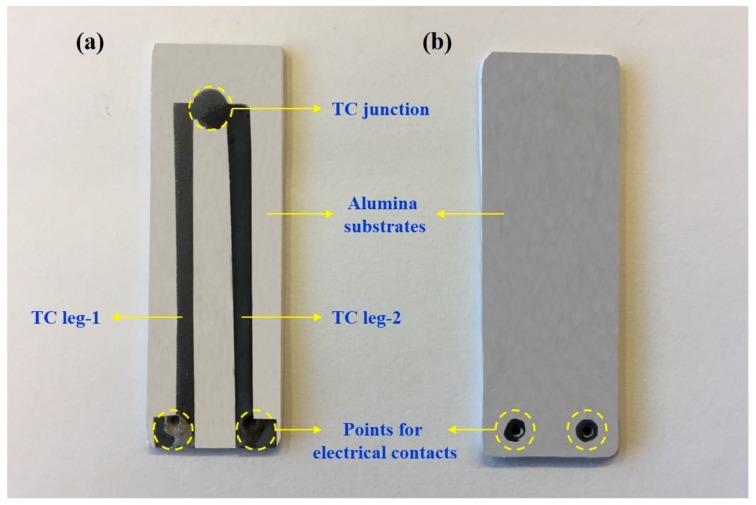
Photograph of a composite thermocouple: (**a**) screen printed on an alumina substrate and sintered at 1500 °C for 2 h, and then (**b**) embedded into alumina preforms.

After composite and platinum ink preparation, the required alumina substrates were prepared by using a tape casting technique. To prepare the ceramic slurries for the tape casting process, deflocculated alumina powders (2.6 m^2^/g SSA; Almatis, Leetsdale, PA, USA) were mixed with polyvinyl butyral binder, poly-alkaline glycol, and benzyl butyl phthalate plasticizers/modifiers (Tape Casting Warehouse, Morrisville, PA, USA) by ball-milling for 24 h using alumina milling media. The as-prepared slurries were then tape casted into thin sheets (~200 µm thickness) and dried at room temperature, which was followed by cutting them into 175 × 175 mm sheets, vacuum packing, and laminating at 90 °C and 10 MPa, respectively. The ~800 µm thick alumina tapes were laser cut to obtain final substrates (30 × 15 × 0.8 mm) for screen printing and thermocouple fabrication. The as-prepared composite inks were screen printed on these alumina substrates by a screen printer (Aremco’s Accu-Coat; Valley Cottage, NY, USA) using a 250-mesh nylon screen. After both left and right legs of thermocouples were printed using the as-prepared metal silicide–alumina ([90–10] MoSi_2_-Al_2_O_3_, [90–10] WSi_2_-Al_2_O_3_, [90–10] TaSi_2_-Al_2_O_3_, [90–10] ZrSi_2_-Al_2_O_3_), and platinum (Pt) inks (depending on thermocouple composition), they were all dried in an oven at 50–60 °C for 5 min. To achieve ~400 µm thick thermocouple legs, these screen printing and drying processes were repeated four times. The composite//Pt and composite//composite thermocouples (~5.1 cm long) screen printed on alumina substrates were lastly sintered at 1500 °C for 2 h under argon atmosphere (50 sccm). All fabricated thermocouple compositions and configurations are presented in Table 1. The **//** symbol used denotes a couple being formed between the compositions preceding and following the symbol. After the optimization of the composite compositions, they were finally embedded into the as-prepared alumina preforms. Figure 1 presents the photograph of a thermocouple screen printed, sintered, and then embedded within alumina preforms.

### 2.2. Structural and Thermoelectric Characterization

The phase development and secondary phase formation of the as-sintered metal silicide–alumina composite thick-film thermocouple legs were analyzed by using an X-ray diffraction instrument (XRD; X’Pert Pro Panalytical, Westborough, MA, USA) with a CuK_α_ radiation source. A field-emission scanning electron microscopy (FE-SEM; Hitachi S-4700F, Tokyo, Japan) was used to investigate their microstructures after sintering. For high-temperature thermoelectric characterization of the as-fabricated composite//Pt and composite//composite thermocouples embedded in alumina preforms, an atmospheric controlled high-temperature furnace was used. Thermoelectric measurements were conducted at temperatures up to 700–1000 °C with increasing thermal gradient by 2 °C/min under argon atmosphere. All electrical contacts were made by using platinum wires and paste at the cold junction. During the measurements, K- and S-type thermocouples were used to record cold and hot junction temperatures with a digital connection from a National Instruments (NI; Austin, TX, USA) thermocouple reader to the computer through LabVIEW software. Thermoelectric voltage (mV) data were acquired using a digital multimeter (Keithley 2100, Tektronix, Beaverton, OR, USA) controlled by the LabVIEW software during the measurements. The thermoelectric voltage output was recorded as point-values due to the stabilized thermal gradient. Seebeck coefficients (S; µV/K) were then calculated by applying the polynomial fitting to the thermoelectric voltage–temperature gradient (mV-ΔT) curves obtained. It is also important to note that multiple samples were tested for each thermocouple configuration. However, the presented thermoelectric results are representation of one sample for each thermocouple configuration, and thus, they do not represent the statistical average values.

## 3. Results

### 3.1. Phase Development and Microstructures of Composites

**Figure 2:**
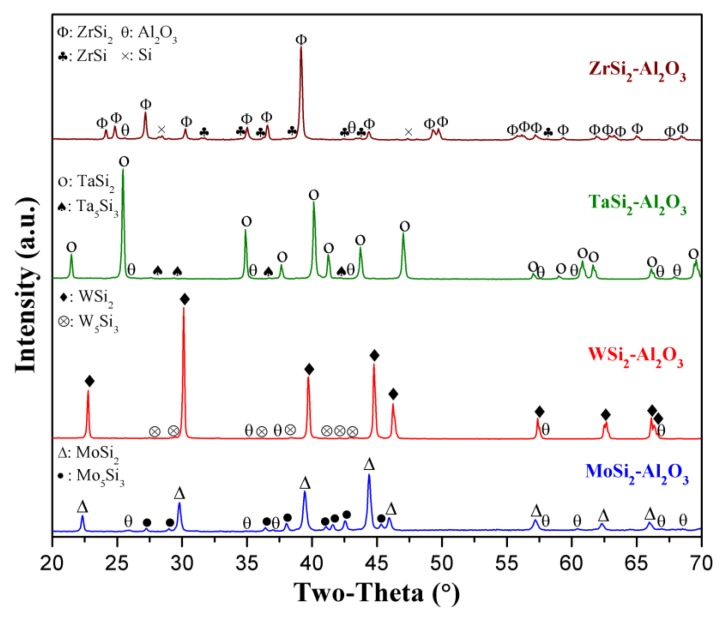
XRD patterns of the [90–10] MoSi_2_-Al_2_O_3_, WSi_2_-Al_2_O_3_, TaSi_2_-Al_2_O_3_, and ZrSi_2_-Al_2_O_3_ composites after sintering at 1500 °C for 2 h.

The XRD phase analysis was completed to investigate the phase development in the various metal silicide–alumina composite systems (thermocouple legs) after sintering at 1500 °C for 2 h. The XRD patterns of the MoSi_2_-Al_2_O_3_, WSi_2_-Al_2_O_3_, TaSi_2_-Al_2_O_3_, and ZrSi_2_-Al_2_O_3_ composites all having [90–10] volume percentages are presented in Figure 2. The XRD peaks corresponding to the starting metal silicides (MoSi_2_, WSi_2_, TaSi_2_, and ZrSi_2_) and alumina phases were detected. Secondary silicide phases were also observed after sintering for all the composite systems. The XRD results revealed that secondary 5-3 metal silicide phases (Mo_5_Si_3_, W_5_Si_3_, Ta_5_Si_3_) were formed during the sintering of the MoSi_2_-Al_2_O_3_, WSi_2_-Al_2_O_3_, and TaSi_2_-Al_2_O_3_ composites. However, the XRD pattern of the ZrSi_2_-Al_2_O_3_ composite showed a different trend, since zirconium monosilicide (ZrSi) and silicon (Si) phases were identified as secondary phases after sintering. The secondary phase formation was found to be related to the interaction of metal silicides with alumina, as well as their reaction with environmental sources such as residual oxygen entrapped in pores or starting silicide powders [15,16,17,18]. In the case of the ZrSi_2_-Al_2_O_3_ system, it may be also correlated to the possible presence of residual zirconium monosilicide (ZrSi) phase in the starting powder and its relatively low melting point (~1500–1520 °C) compared to that of ZrSi_2_ (1620 °C) [19]. Since the melting point of ZrSi is close to the sintering temperature, it could result in partial melting during the sintering hold at temperature, and thus, a slight formation of the silicon (Si) phase as detected by XRD. The presence of ZrSi and Si phases along with the ZrSi_2_ phase was similarly found in the Zr/Si diffusion couple after high-temperature annealing at 1200 °C [20]. Furthermore, Rietveld refinement results in the authors’ previous study [14] demonstrated that ~20.2 vol % of a secondary Mo_5_Si_3_ phase formed in the [90–10] MoSi_2_-Al_2_O_3_ composite after sintering. Studies reported that the presence of the metal silicide phases with 5-3 stoichiometry could be also beneficial for high-temperature applications due to their higher melting points, good thermal stability, high resistance to creep, and sufficient mechanical strength [21,22,23]. It is important to point out that these secondary phases could significantly affect not only the thermoelectric properties of the fabricated ceramic composite thermocouples, but also their stability and mechanical and thermal properties at elevated temperatures.

**Figure 3:**
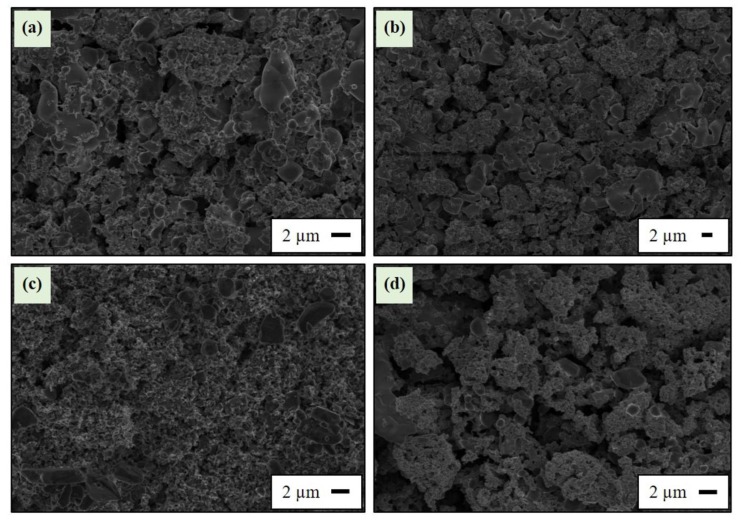
SEM micrographs of the [90–10] (**a**) MoSi_2_-Al_2_O_3_, (**b**) WSi_2_-Al_2_O_3_, (**c**) TaSi_2_-Al_2_O_3_, and (**d**) ZrSi_2_-Al_2_O_3_ composites after sintering at 1500 °C for 2 h.

Figure 3 shows the SEM microstructures of the MoSi_2_-Al_2_O_3_, WSi_2_-Al_2_O_3_, TaSi_2_-Al_2_O_3_, and ZrSi_2_-Al_2_O_3_ composites all with [90–10] volume percentages after sintering at 1500 °C for 2 h. No significant contrast was achieved to differentiate silicide and alumina particles. It is known from the authors’ previous studies [15,21] that these metal silicides have random morphology with 4.2–6.4 µm average particle size, whereas alumina particles have equiaxed morphology and an average size of 0.4 µm. Therefore, the agglomeration of large (silicide) and small (alumina) grains was evident in certain regions for all composite microstructures. This could not adversely affect the electrical percolation network due to the presence of a high metal silicide concentration (90 vol %). A certain level of porosity within all composite microstructures can be also seen. In addition, it is important to note that good adhesion was achieved between screen-printed thermocouple legs (composite//Pt and composite//composite) and the alumina substrate after sintering at 1500 °C. No interfacial microcracking was observed within any composite microstructures. It could be directly interrelated with the close thermal expansion coefficient match between these metal silicides (MoSi_2_: 8.2 × 10^−6^ K^−1^, WSi_2_: 7.9 × 10^−6^ K^−1^, ZrSi_2_: 8.3 × 10^−6^ K^−1^, TaSi_2_: 8.9 × 10^−6^ K^−1^) and Al_2_O_3_ (~8.0 × 10^−6^ K^−1^) in a wide temperature region [19]. This is one of the major benefits of using silicide–alumina composites for high-temperature and harsh-environment sensing.

### 3.2. Thermoelectric Properties of Composite Thermocouples

**Figure 4:**
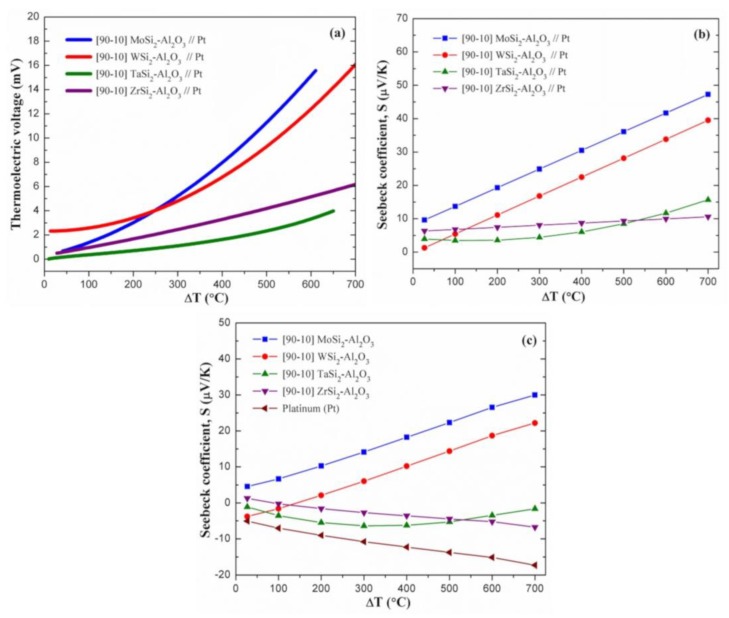
(**a**) Thermoelectric voltage and (**b**) effective Seebeck coefficients (S) measured for the [90–10] MoSi_2_-Al_2_O_3_//Pt, WSi_2_-Al_2_O_3_//Pt, TaSi_2_-Al_2_O_3_//Pt, and ZrSi_2_-Al_2_O_3_//Pt thermocouples as a function of temperature difference; and (**c**) calculated intrinsic Seebeck coefficients of the composites as a function of temperature difference (S_Pt_ is also presented as a reference [26]).

Prior to thermoelectric characterization of the composite//composite thermocouples, high-temperature thermoelectric properties of the composite//Pt thermocouples were examined to better understand the intrinsic Seebeck coefficient of each composite. Figure 4a displays the thermoelectric voltage (E in mV) of the MoSi_2_-Al_2_O_3_//Pt, WSi_2_-Al_2_O_3_//Pt, TaSi_2_-Al_2_O_3_//Pt, and ZrSi_2_-Al_2_O_3_//Pt thermocouples as a function of temperature gradient (ΔT in °C). It is clear that the thermoelectric voltage of all composite//Pt thermocouples increased with increasing temperature gradient, but at different levels. At ΔT = 500 °C, MoSi_2_-Al_2_O_3_//Pt and WSi_2_-Al_2_O_3_//Pt thermocouples generated 11.3 and 9.3 mV thermoelectric voltage, respectively. These values were found to be substantially higher than that of the TaSi_2_-Al_2_O_3_//Pt (2.3 mV) and ZrSi_2_-Al_2_O_3_//Pt (4.2 mV) thermocouples. It is also evident that the MoSi_2_-Al_2_O_3_//Pt thermocouple exhibited higher thermoelectric voltage than other composite//Pt thermocouples when the temperature gradient was above 260 °C. On the other hand, the TaSi_2_-Al_2_O_3_//Pt thermocouple displayed thermoelectric voltage less than 2.0 mV at ΔT ≤ 455 °C, which was significantly lower compared to the other composite//Pt thermocouples. Figure 4b shows the effective Seebeck coefficients (S) of these composite//Pt thermocouples, which were calculated by applying the second- or third-order polynomial fitting to the thermoelectric voltage–temperature gradient (E-ΔT) curves and then using their first derivatives (slopes) as shown in the equations below based on the theory (slope method) [24,25]:

2nd order → E = A·ΔT + B·ΔT^2^ + C → S = A + 2B·ΔT (1)

3rd order → E = A·ΔT + B·ΔT^2^ + D·ΔT^3^ + C → S = A + 2B·ΔT + 3D·ΔT^2^ (2)

The fitting coefficients (A, B, and D), which were achieved via polynomial fitting and then used for Seebeck coefficient calculations, are additionally listed in Table 2. Prior to these calculations, units of fitting coefficients (including mV) were converted to calculate the Seebeck coefficients in µV/K. The effective Seebeck coefficient of the [90–10] MoSi_2_-Al_2_O_3_//Pt thermocouple increased from 9.6 to 47.3 µV/K with increasing temperature gradient (27 °C → 700 °C). This thermocouple displayed higher Seebeck coefficients than other composite//Pt thermocouples throughout ΔT range. In addition, [90–10] WSi_2_-Al_2_O_3_//Pt thermocouples revealed a similar thermoelectric behavior, since its effective Seebeck coefficient ranged between 1.3 and 39.5 µV/K. However, relatively low Seebeck coefficients were achieved by [90–10] ZrSi_2_-Al_2_O_3_//Pt and [90–10] TaSi_2_-Al_2_O_3_//Pt thermocouples, particularly at the temperature gradients above 200 °C. The effective Seebeck coefficient of the [90–10] ZrSi_2_-Al_2_O_3_//Pt thermocouple increased from 6.3 to 10.6 µV/K with increasing temperature gradient, whereas this increase was from 3.9 to 15.7 µV/K for the [90–10] TaSi_2_-Al_2_O_3_//Pt thermocouple. It is evident that the level of increase in the Seebeck coefficients as a function of temperature gradient was much lower for these thermocouples compared to the MoSi_2_-Al_2_O_3_//Pt and WSi_2_-Al_2_O_3_//Pt thermocouples. At 500 °C, the effective Seebeck coefficients of the [90–10] MoSi_2_-Al_2_O_3_//Pt, WSi_2_-Al_2_O_3_//Pt, TaSi_2_-Al_2_O_3_//Pt, and ZrSi_2_-Al_2_O_3_//Pt thermocouples were measured as 36.1, 28.2, 8.5, and 9.3 µV/K, respectively. Furthermore, [90–10] ZrSi_2_-Al_2_O_3_//Pt displayed the lowest effective Seebeck coefficients at temperature gradients above ~550 °C.

The experimental effective Seebeck coefficient data for the composite//Pt thermocouples (S_composite//Pt_) were used to obtain the calculated intrinsic Seebeck coefficients of the metal silicide–oxide composites. For these calculations, the measured temperature-dependent Seebeck coefficient of a platinum wire (S_Pt_ ranging from −5.1 to −17.3 µV/K) was used as a reference from the literature [26]. The intrinsic Seebeck coefficients of silicide–oxide composites (S_composite_) were calculated using the equation below:

S_composite//Pt_ = S_composite_−S_Pt_ (3)

Figure 4c presents the calculated intrinsic Seebeck coefficients of the silicide–oxide composites (S_composite_), which were calculated as described above. The reference Seebeck coefficient data of platinum (S_Pt_) were also shown within the figure. [90–10] MoSi_2_-Al_2_O_3_ and [90–10] WSi_2_-Al_2_O_3_ composites showed an increasing linear trend for their intrinsic Seebeck coefficients with increasing temperature gradient. The intrinsic Seebeck coefficient of the [90–10] MoSi_2_-Al_2_O_3_ composite was found to range between 4.6 and 30.0 µV/K at ΔT range of 27–700 °C. This increase was from −3.8 to 22.2 µV/K for the [90–10] WSi_2_-Al_2_O_3_ composite. It is clear that the MoSi_2_-Al_2_O_3_ composite displayed a higher intrinsic Seebeck coefficient than other silicide–oxide composites throughout the ΔT range. In addition, the WSi_2_-Al_2_O_3_ composite revealed negative Seebeck coefficients at the temperature gradients lower than ~145 °C, whereas MoSi_2_-Al_2_O_3_ always generated positive Seebeck coefficients. The Seebeck coefficients of MoSi_2_ and WSi_2_ were reported as −5.4 and −0.4 µV/K at room temperature [7]. It is evident that the calculated Seebeck coefficients of [90–10] MoSi_2_-Al_2_O_3_ (4.6 µV/K at 27 °C) and [90–10] WSi_2_-Al_2_O_3_ (−3.8 µV/K at 27 °C) composites differ from these reported values. This could be related to a certain presence of Mo_5_Si_3_ and W_5_Si_3_ secondary phases within the composite structures after sintering. As discussed earlier, the amount of Mo_5_Si_3_ phase within the [90–10] MoSi_2_-Al_2_O_3_ composite was determined as 20.2 vol % using the Rietveld method. These results may indicate that the intrinsic Seebeck coefficients of Mo_5_Si_3_ and W_5_Si_3_ could have positive and negative signs at room temperature, respectively. However, no study was found on the Seebeck coefficients of these 5-3 intermetallic phases, and thus, future studies are needed. Additionally, the thermoelectric output of the MoSi_2_ thin-film thermocouple at 500 °C ranged between 19.4 and 64.1 µV/°C, depending on the heat treatment conditions [6]. On the other hand, the [90–10] ZrSi_2_-Al_2_O_3_ composite exhibited a decreasing linear trend (Figure 4c). This decrease was determined to be from 1.3 to −6.7 µV/K with increasing temperature gradient. For the [90–10] TaSi_2_-Al_2_O_3_ composite, two different regimes were observed. Its intrinsic Seebeck coefficient firstly increased in negative sign from −1.1 to −6.4 µV/K with increasing ΔT from 27 °C to 300 °C, and then, decreased to −1.6 µV/K with increasing temperature gradient to 700 °C. The Seebeck coefficient of TaSi_2_ was reported as 25.0 µV/K at room temperature [7], which is highly different from that of the TaSi_2_-Al_2_O_3_ composite in this study. This could be due to the formation of Ta_5_Si_3_ phase, which may have a negative intrinsic Seebeck coefficient at the same temperature. Similarly, no data were reported on thermoelectric properties of Ta_5_Si_3_ and ZrSi secondary phases. As a result, it should be noted that the calculated intrinsic Seebeck coefficients of [90–10] silicide–oxide composites relatively differ from the reported thermoelectric data of these silicides, which demonstrates that secondary silicide phases could have significantly different intrinsic thermoelectric properties than their disilicide forms. Based on the calculated values, MoSi_2_-Al_2_O_3_ and WSi_2_-Al_2_O_3_ composites showed the highest (and also positive) intrinsic Seebeck coefficients at high temperatures. The intrinsic Seebeck coefficients of ZrSi_2_-Al_2_O_3_ and TaSi_2_-Al_2_O_3_ composites were calculated to be relatively low, and they both showed a negative value at high temperatures.

**Figure 5:**
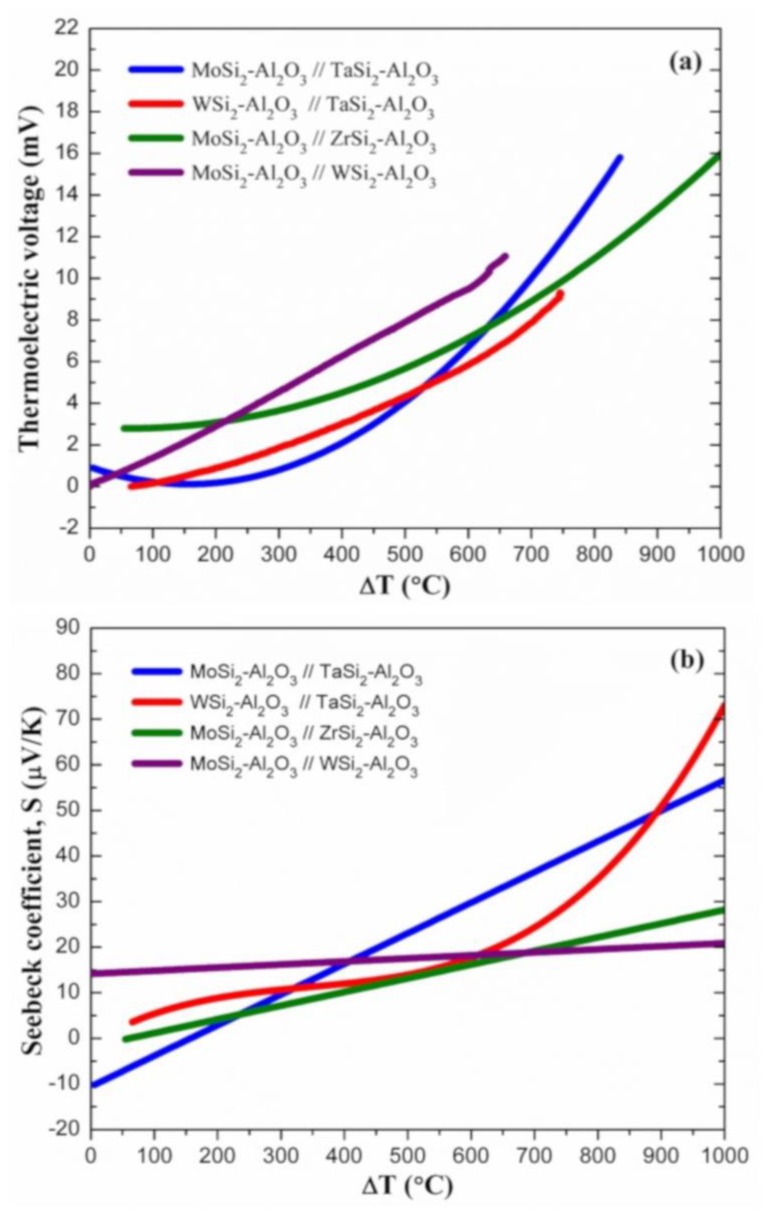
(**a**) Thermoelectric voltage and (**b**) effective Seebeck coefficients measured for the MoSi_2_-Al_2_O_3_//TaSi_2_-Al_2_O_3_, WSi_2_-Al_2_O_3_//TaSi_2_-Al_2_O_3_, MoSi_2_-Al_2_O_3_//ZrSi_2_-Al_2_O_3_, and MoSi_2_-Al_2_O_3_//WSi_2_-Al_2_O_3_ composite thermocouples as a function of temperature difference (composite volume percentages were fixed at [90–10] on both legs of the thermocouples).

After studying the thermoelectric properties of composite//Pt thermocouples and silicide–oxide composites, various composite//composite thermocouples were also fabricated and tested. Similar to the composite//Pt thermocouples, all composite//composite thermocouples were made of [90–10] vol % silicide–oxide composites. The thermoelectric voltage and effective Seebeck coefficients of composite//composite thermocouples are presented as a function of temperature gradient in Figure 5. The thermoelectric voltage of all composite//composite thermocouples similarly increased with increasing temperature gradient. At ΔT = 500 °C, the MoSi_2_-Al_2_O_3_//WSi_2_-Al_2_O_3_ thermocouple generated 7.9 mV, which is higher than the thermoelectric output of other composite//composite thermocouples. At the same ΔT, the MoSi_2_-Al_2_O_3_//ZrSi_2_-Al_2_O_3_ thermocouple exhibited a thermoelectric voltage of 5.7 mV. However, thermoelectric outputs of MoSi_2_-Al_2_O_3_//TaSi_2_-Al_2_O_3_ and WSi_2_-Al_2_O_3_//TaSi_2_-Al_2_O_3_ thermocouples were found to be similar, but relatively lower (4.1–4.3 mV) compared to other composite//composite thermocouples. The thermoelectric performance of MoSi_2_-Al_2_O_3_//TaSi_2_-Al_2_O_3_ and MoSi_2_-Al_2_O_3_//ZrSi_2_-Al_2_O_3_ thermocouples was further characterized at higher temperature gradients (Figure 5a). At ΔT = 840 °C, the MoSi_2_-Al_2_O_3_//TaSi_2_-Al_2_O_3_ composite thermocouple generated 15.8 mV, which is significantly higher than that of the MoSi_2_-Al_2_O_3_//ZrSi_2_-Al_2_O_3_ thermocouple (11.9 mV). In addition, the MoSi_2_-Al_2_O_3_//ZrSi_2_-Al_2_O_3_ thermocouple revealed a thermoelectric voltage of 16.0 mV at ΔT = 1000 °C. When considering these mV-ΔT curves, it is evident that the MoSi_2_-Al_2_O_3_//TaSi_2_-Al_2_O_3_ thermocouple performs better than other composite//composite thermocouples at higher temperature gradients (ΔT ≥ ~800 °C). Figure 5b shows the effective Seebeck coefficients of the composite//composite thermocouples, which were similarly calculated by utilizing the second- or fourth-order (Equation 4) polynomial fitting to the thermoelectric voltage–temperature gradient (E-ΔT) curves and then using their first derivatives:

4th order → E = A·ΔT + B·ΔT^2^ + D·ΔT^3^ + E·ΔT^4^ + C → S = A + 2B·ΔT + 3D·ΔT^2^ + 4E·ΔT^3^ (4)

The polynomial fitting coefficients (A, B, D, and E) are also listed in Table 2. These coefficients (slope method) were further used to calculate their effective Seebeck coefficients up to a temperature gradient of 1000 °C. The effective Seebeck coefficient of the MoSi_2_-Al_2_O_3_//WSi_2_-Al_2_O_3_ thermocouple increased from 14.4 to 20.9 µV/K with increasing ΔT from 27 °C to 1000 °C. It is determined that this thermocouple displayed higher Seebeck coefficients (14.2–16.9 µV/K) than all other composite//composite thermocouples at ΔT up to ~400 °C. A significant increase in the effective Seebeck coefficients of MoSi_2_-Al_2_O_3_//TaSi_2_-Al_2_O_3_ and WSi_2_-Al_2_O_3_//TaSi_2_-Al_2_O_3_ thermocouples was observed with increasing ΔT (Figure 5b). The effective Seebeck coefficients of the MoSi_2_-Al_2_O_3_//TaSi_2_-Al_2_O_3_ thermocouple were all negative at ΔT ≤ ~155 °C, whereas it increased to 56.7 µV/K with increasing ΔT to 1000 °C. It also displayed higher Seebeck coefficients than other composite//composite thermocouples at ΔT range of ~400–900 °C. It was also found that the effective Seebeck coefficient of the WSi_2_-Al_2_O_3_//TaSi_2_-Al_2_O_3_ thermocouple drastically increased from 17.7 to 73.0 µV/K with increasing ΔT from 600 °C to 1000 °C. At ΔT > 900 °C, this thermocouple clearly exhibited the highest Seebeck coefficients. In addition, the MoSi_2_-Al_2_O_3_//ZrSi_2_-Al_2_O_3_ thermocouple revealed an increasing linear trend, where its effective Seebeck coefficient increased from 0.1 to 28.1 µV/K with increasing ΔT.

Lastly, the effective Seebeck coefficients of composite//composite thermocouples were additionally calculated using the previously calculated intrinsic Seebeck coefficients of [90–10] vol % silicide–oxide composites, which were previously presented in Figure 4c. The equation is listed below for these calculations based on the Seebeck theory:

S_composite #1_−S_composite #2_ = S_composite #1//composite #2_ (5)

**Figure 6:**
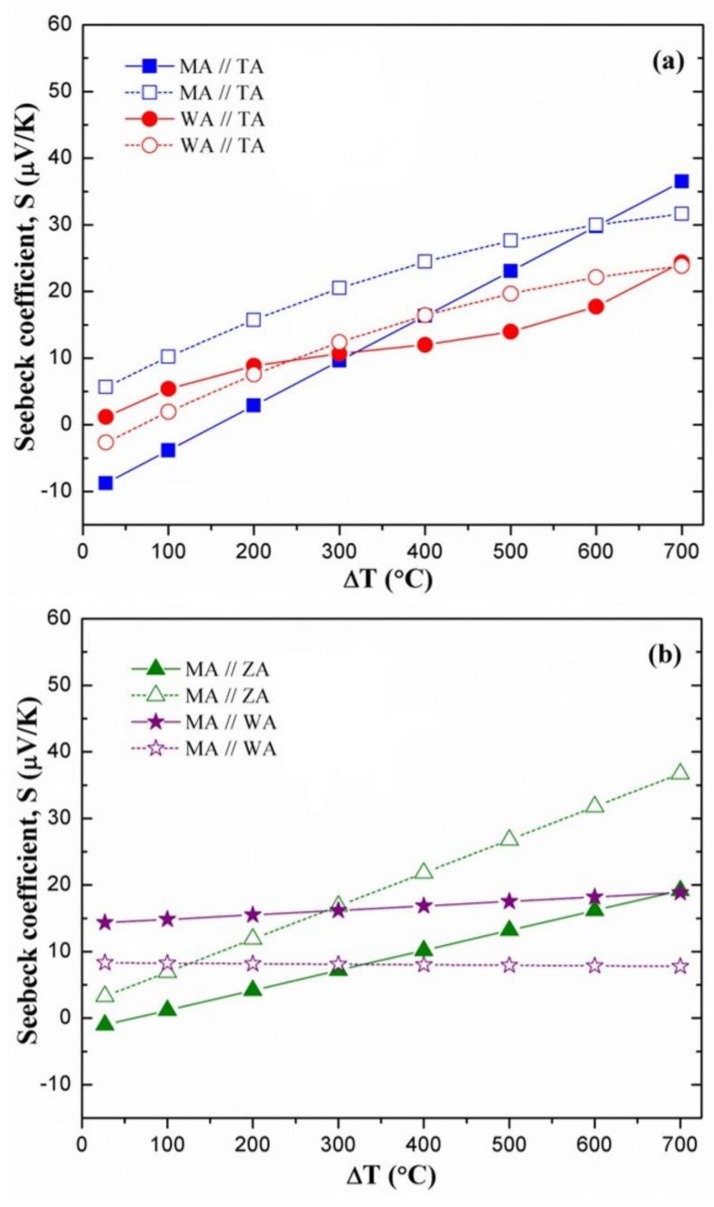
Comparison of the calculated (dashed lines) and measured effective (solid lines) Seebeck coefficients of the (**a**) MoSi_2_-Al_2_O_3_//TaSi_2_-Al_2_O_3_ and WSi_2_-Al_2_O_3_//TaSi_2_-Al_2_O_3_, and (**b**) MoSi_2_-Al_2_O_3_//ZrSi_2_-Al_2_O_3_ and MoSi_2_-Al_2_O_3_//WSi_2_-Al_2_O_3_ composite thermocouples as a function of temperature difference (composites were abbreviated as MA//TA, WA//TA, MA//ZA, and MA//WA, respectively).

The composite and related thermocouple compositions were also abbreviated for simplicity (MA: MoSi_2_-Al_2_O_3_, WA: WSi_2_-Al_2_O_3_, TA: TaSi_2_-Al_2_O_3_, ZA: ZrSi_2_-Al_2_O_3_). For example; the Seebeck coefficient of a MoSi_2_-Al_2_O_3_//TaSi_2_-Al_2_O_3_ (S_MA//TA_) composite thermocouple was calculated by subtracting the calculated intrinsic Seebeck coefficient of the TaSi_2_-Al_2_O_3_ composite (S_TA_) from that of the MoSi_2_-Al_2_O_3_ composite (S_MA_). The comparison of the calculated and measured effective Seebeck coefficients of composite//composite thermocouples is presented as a function of temperature gradient (27–700 °C) in Figure 6. The measured Seebeck coefficients of MoSi_2_-Al_2_O_3_//ZrSi_2_-Al_2_O_3_ (MA//ZA) and MoSi_2_-Al_2_O_3_//TaSi_2_-Al_2_O_3_ (MA//TA) thermocouples were found to be mostly lower than their calculated Seebeck coefficients. However, at ΔT > 500 °C, the calculated and measured values were similar for the MoSi_2_-Al_2_O_3_//TaSi_2_-Al_2_O_3_ thermocouple. On the contrary, the measured Seebeck coefficients of the MoSi_2_-Al_2_O_3_//WSi_2_-Al_2_O_3_ (MA//WA) thermocouple (14.4–18.9 µV/K) were higher than the calculated values (7.8–8.3 µV/K). Such variations could be due to the certain presence of secondary phases within the composite systems. These results may indicate that the overall thermoelectric effect of molybdenum-silicide phases (MoSi_2_, Mo_5_Si_3_) with respect to zirconium- and tantalum-silicide phases (ZrSi_2_, ZrSi, TaSi_2_, Ta_5_Si_3_) may adversely influence the thermoelectric performance of MoSi_2_-Al_2_O_3_//ZrSi_2_-Al_2_O_3_ and MoSi_2_-Al_2_O_3_//TaSi_2_-Al_2_O_3_ thermocouples. However, the higher measured Seebeck coefficients for the MoSi_2_-Al_2_O_3_//WSi_2_-Al_2_O_3_ thermocouple could imply the positive influence of different silicide couples (MoSi_2_-W_5_Si_3_, Mo_5_Si_3_-WSi_2_, Mo_5_Si_3_-W_5_Si_3_) on its thermoelectric output. On the other hand, the measured and calculated Seebeck coefficients of the WSi_2_-Al_2_O_3_//TaSi_2_-Al_2_O_3_ (WA//TA) thermocouple displayed a very close match throughout the ΔT range. These results also pointed out that the thermoelectric performance of composite//composite thermocouples may be positively or negatively affected by possible local compositional changes at the junction, which need to be further investigated in detail.

As presented in Figure 6, the calculated and measured Seebeck coefficients of the MoSi_2_-Al_2_O_3_//WSi_2_-Al_2_O_3_ (MA//WA) thermocouple were 8.3 and 14.4 µV/K at ΔT = 27 °C, respectively. The effective Seebeck coefficient of a MoSi_2_//WSi_2_ thermocouple (without the addition of Al_2_O_3_) could be estimated as 5.0 µV/K at room temperature from the previously reported data [7]. Therefore, it is evident that its calculated Seebeck coefficient is relatively close to this theoretical estimation, but its measured Seebeck coefficient is substantially higher, indicating the positive influence of secondary 5-3 silicide phases (Mo_5_Si_3_, W_5_Si_3_) on overall thermoelectric performance. The calculated and measured Seebeck coefficients of the MoSi_2_-Al_2_O_3_//TaSi_2_-Al_2_O_3_ (MA//TA) thermocouple were 5.7 and −8.7 µV/K at ΔT = 27 °C, respectively. At the same temperature gradient, the WSi_2_-Al_2_O_3_//TaSi_2_-Al_2_O_3_ (WA//TA) thermocouple displayed calculated and measured Seebeck coefficients of −2.7 and 1.2 µV/K, respectively. From the reported data [7], the effective Seebeck coefficients of MoSi_2_//TaSi_2_ and WSi_2_//TaSi_2_ thermocouples should be near 30.4 and 25.4 µV/K at room temperature, respectively. These theoretical values are significantly higher than the calculated and measured Seebeck coefficients of these composite//composite (MA//TA and WA//TA) thermocouples. This indicates that the intrinsic Seebeck coefficient of Ta_5_Si_3_ secondary phase may be greatly lower than that of tantalum disilicide (TaSi_2_), which could negatively affect the thermoelectric performance of these composite//composite thermocouples. Similar comparisons were not used for the MoSi_2_-Al_2_O_3_//ZrSi_2_-Al_2_O_3_ (MA//ZA) thermocouple due to the lack of intrinsic Seebeck coefficient data for ZrSi_2_ phase in literature. In brief, all composite//composite thermocouples exhibited sufficiently high sensitivity (ranging from −8.7 to 73.0 µV/K) at the range of 27–1000 °C. In a similar temperature range, it was reported for the mostly used high-temperature thermocouples that S-type (90% Pt-10% Rh//Pt) and R-type (87% Pt-13% Rh//Pt) thermocouples possess 5.5–13.0 µV/K sensitivity [27]. They also showed higher Seebeck coefficients than platinum//palladium (Pt//Pd) thin-film thermocouples, which displayed performances near −12.9 and −14.3 µV/°C at 900 °C [3]. Furthermore, in the authors’ previous study, the thermoelectric characterization of a long MoSi_2_-Al_2_O_3_//WSi_2_-Al_2_O_3_ composite thermocouple demonstrated its highly stable thermoelectric response with no drift in voltage output during a 10 h isothermal hold at 1350 °C [14]. It is highly evident that the thermoelectric performance and the sensitivity of these silicide–oxide-based ceramic composite thermocouples are very promising for high-temperature sensing under harsh environment conditions, and also highly advantageous and cost effective in comparison to precious metal-based temperature sensors.

## 4. Conclusions

The fabrication and high-temperature thermoelectric properties of composite//Pt and composite//composite thick-film ceramic thermocouples were examined by using various metal silicide–alumina composites. The intrinsic Seebeck coefficients of silicide–alumina composites were calculated from the measured effective Seebeck coefficients of composite//Pt thermocouples. The calculated intrinsic Seebeck coefficients of MoSi_2_-Al_2_O_3_ and WSi_2_-Al_2_O_3_ composites at high temperatures (22.2–30.0 µV/K) were found to be higher than that of other composites. The composite//composite thermocouples generated a thermoelectric voltage up to 15.8–16.0 mV at high temperatures. Their effective Seebeck coefficients at a temperature gradient of 1000 °C were found to range between 20.9 and 73.0 µV/K. It was determined that the measured effective Seebeck coefficients of composite//composite thermocouples differ from the calculated values. These results clearly indicated the major positive or negative influence of secondary phases and their Seebeck coefficients with respect to other disilicide and secondary phases on their overall thermoelectric response. In addition, these composite//composite thermocouples displayed stable thermoelectric performance, and also higher sensitivity than the commonly used precious metal-based thermocouples. Therefore, metal silicide–alumina composite-based thermocouples could be promising materials with stable performance, high sensitivity, and lower cost for high-temperature sensing under harsh environments, and they may potentially serve as a replacement of expensive precious metal-based thermocouples in the near future. However, it should be noted that further studies are needed such as examining the intrinsic thermoelectric properties of other silicide phases (e.g., Mo_5_Si_3_, Ta_5_Si_3_, ZrSi_2_) and their influence on the thermoelectric properties of such thermocouples, as well as characterizing the thermocouple junctions to better understand their thermoelectric response. Future studies are also essential for understanding their thermoelectric performance and lifetime and potential drift aspects under both argon and oxidizing conditions.

## Figures and Tables

**Table 1:** Configurations and compositions of the silicide–alumina//Pt and silicide–alumina// silicide–alumina thermocouples studied (volume percentages are represented by [ ]).

Thermocouple	Leg 1	Leg 2
[90–10] MoSi_2_-Al_2_O_3_//Pt	[90–10] MoSi_2_-Al_2_O_3_	Pt
[90–10] WSi_2_-Al_2_O_3_//Pt	[90–10] WSi_2_-Al_2_O_3_	Pt
[90–10] TaSi_2_-Al_2_O_3_//Pt	[90–10] TaSi_2_-Al_2_O_3_	Pt
[90–10] ZrSi_2_-Al_2_O_3_//Pt	[90–10] ZrSi_2_-Al_2_O_3_	Pt
[90–10] MoSi_2_-Al_2_O_3_//[90–10] TaSi_2_-Al_2_O_3_	[90–10] MoSi_2_-Al_2_O_3_	[90–10] TaSi_2_-Al_2_O_3_
[90–10] WSi_2_-Al_2_O_3_//[90–10] TaSi_2_-Al_2_O_3_	[90–10] WSi_2_-Al_2_O_3_	[90–10] TaSi_2_-Al_2_O_3_
[90–10] MoSi_2_-Al_2_O_3_//[90–10] ZrSi_2_-Al_2_O_3_	[90–10] MoSi_2_-Al_2_O_3_	[90–10] ZrSi_2_-Al_2_O_3_
[90–10] MoSi_2_-Al_2_O_3_//[90–10] WSi_2_-Al_2_O_3_	[90–10] MoSi_2_-Al_2_O_3_	[90–10] WSi_2_-Al_2_O_3_

**Table 2:** List of polynomial fitting coefficients used for thermoelectric calculations.

Thermocouple	A (µV/K)	B (µV/K^2^)	D (µV/K^3^)	E (µV/K^4^)	Adj. R^2^
[90–10] MoSi_2_-Al_2_O_3_//Pt	8.09	0.0280	-	-	1.000
[90–10] WSi_2_-Al_2_O_3_//Pt	−0.25	0.0284	-	-	1.000
[90–10] TaSi_2_-Al_2_O_3_//Pt	4.20	−0.0056	1.31 × 10^−5^	-	0.999
[90–10] ZrSi_2_-Al_2_O_3_//Pt	6.20	0.0032	-	-	1.000
[90–10] MoSi_2_-Al_2_O_3_//[90-10] TaSi_2_-Al_2_O_3_	−10.55	0.0336	-	-	1.000
[90–10] WSi_2_-Al_2_O_3_//[90-10] TaSi_2_-Al_2_O_3_	−0.85	0.0400	−6.51 × 10^−5^	4.73 × 10^−8^	1.000
[90–10] MoSi_2_-Al_2_O_3_//[90-10] ZrSi_2_-Al_2_O_3_	−1.82	0.0150	-	-	1.000
[90–10] MoSi_2_-Al_2_O_3_//[90-10] WSi_2_-Al_2_O_3_	14.17	0.0033	-	-	0.999
